# Umbilical port hernia following laparoscopic cholecystectomy

**DOI:** 10.4103/0972-9941.25675

**Published:** 2006-03

**Authors:** P Singh, R Kaushik, R Sharma

**Affiliations:** Department of Surgery, Government Medical College and Hospital, Chandigarh, Haryana, India

The incidence of incisional hernia occurring at the port sites after laparoscopic surgery, lies between 0.02 to 3.6%[1] and usually remains unreported, until the development of complications.[2] Such a case is reported here and the problem briefly reviewed.

A 55 year-old obese lady presented to the emergency ward, with the complaints of pain, redness and swelling of the periumbilical region, for a duration of 4 days. She had undergone laparoscopic cholecystectomy at our hospital 4 years ago and had remained well, prior to the present problem. Examination revealed cellulitis of the periumbilical area with induration and a vague swelling without cough impulse. The local temperature was also elevated. When directly questioned, she was not aware of any hernia at the site, either before or after laparoscopic cholecystectomy. Her routine hematological and biochemical investigations were within normal limits. Contract enhanced computerised tomogram (CECT) scan revealed a defect in the midline at the level of the umbilicus, with herniation of the intra-abdominal contents through this defect [Figure 1]. With a diagnosis of port site hernia, she was taken up for surgery.

**Figure 1:**
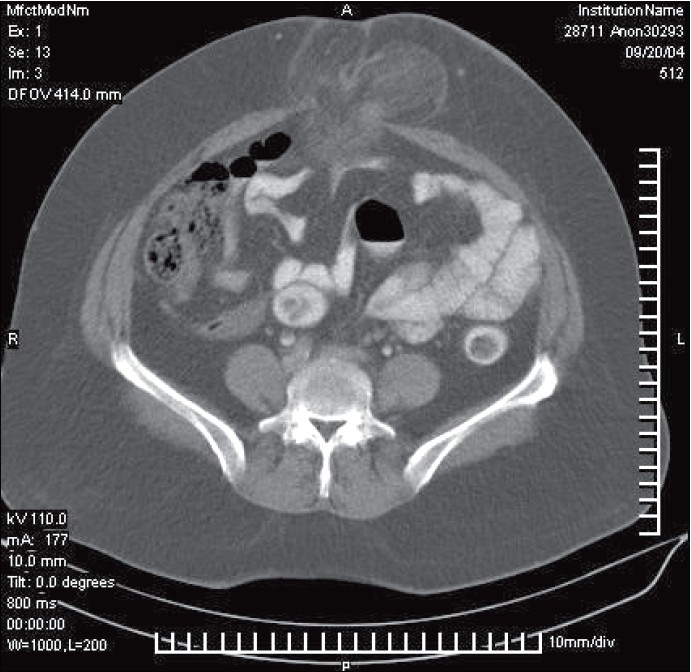
CECT of the abdomen showing herniation of the omentum through the umbilical port site

Surgery was performed under general anaesthesia and revealed herniation of the omentum through the port site. There were dense adhesions and areas of gangrene in the herniated omentum. The gangrenous parts were excised and the rest of the omentum reposited back into the abdomen. Closure was performed using *0* polypropylene suture and reinforced with an onlay mesh. The patient remained well in the post-operative period and was discharged on the 5^th^ post-operative day in a satisfactory condition.

Incisional herniae can occur after any abdominal surgery and laparoscopic surgery is not immune to this complication. The herniae that follow laparoscopy usually occur through the larger ports (size greater than 10 mm), especially the umbilicus,[3] but the smaller ports can also be affected. Various factors have been implicated in their development - wound extension, male sex, infection of the wound, pre-existing umbilical defects, post-operative chest infections and pre-existing diseases such as diabetes mellitus and connective tissue disorders; but the single most important factor in their development remains the improper closure of the fascial defects at the port sites.[4,5]

The hernia may become evident at any time following laparoscopic surgery and the patient may either have an uncomplicated hernia, or may be afflicted with a variety of complications such as evisceration of the bowel or omentum and it may become a cause of significant morbidity[3] Meticulous closure of the fascia, avoidance of unnecessary wound extension, the use of non-absorbable sutures when faced with defects more than 2 cms in size, completely defining the extent of any pre-existing hernia and repairing this at the time of port site closure, are recommended to minimize the incidence of port site herniae after laparoscopic surgery.[4,5]

## References

[1] Bergemann JL, Hibbert ML, Harkins G, Narvaez J, Asato A (2001). Omental herniation through a 3-mm umbilical trocar site:unmasking a hidden umbilical hernia. J Laparoendosc Adv Surg Tech A.

[2] Yuen PM (1995). Early incisional hernia following laparoscopic surgery. Aust N Z J Obstet Gynaecol.

[3] Azurin DJ, Go LS, Arroyo LR, Kirkland ML (1995). Trocar site herniation following laparoscopic cholecystectomy and the significance of an incidental preexisting umbilical hernia. Am Surg.

[4] Nassar AH, Akshar KA, Rashed AA, Abdulmoneum MG (1997). Laparoscopic cholecystectomy and the umbilicus. Br J Surg.

[5] Ramachandran CS (1998). Umbilical hernial defects encountered before and after abdominal laparoscopic procedures. Int Surg.

